# Steady-state and dynamic network modes for perceptual expectation

**DOI:** 10.1038/srep40626

**Published:** 2017-01-12

**Authors:** Uk-Su Choi, Yul-Wan Sung, Seiji Ogawa

**Affiliations:** 1 Neuroscience Research Institute, Gachon University of Medicine and Science, Incheon, Republic of Korea; 2 Kansei Fukushi Research Institute, Tohoku Fukushi University, Sendai, Japan

## Abstract

Perceptual expectation can attenuate repetition suppression, the stimulus-induced neuronal response generated by repeated stimulation, suggesting that repetition suppression is a top-down modulatory phenomenon. However, it is still unclear which high-level brain areas are involved and how they interact with low-level brain areas. Further, the temporal range over which perceptual expectation can effectively attenuate repetition suppression effects remains unclear. To elucidate the details of this top-down modulatory process, we used two short and long inter-stimulus intervals for a perceptual expectation paradigm of paired stimulation. We found that top-down modulation enhanced the response to the unexpected stimulus when repetition suppression was weak and that the effect disappeared at 1,000 ms prior to stimulus exposure. The high-level areas involved in this process included the left inferior frontal gyrus (IFG_L) and left parietal lobule (IPL_L). We also found two systems providing modulatory input to the right fusiform face area (FFA_R): one from IFG_L and the other from IPL_L. Most importantly, we identified two states of networks through which perceptual expectation modulates sensory responses: one is a dynamic state and the other is a steady state. Our results provide the first functional magnetic resonance imaging (fMRI) evidence of temporally nested networks in brain processing.

Repetition suppression of brain responses observed using functional magnetic resonance imaging (fMRI) reflects short-term neuronal plasticity that results from sensitization or fatigue of neuronal circuits^1^. This process involves the adaptation of populations of neurons responding to the stimulus and occurs via a reduction in activation levels, number of neurons, or processing time^1^. The repetition suppression phenomenon has been used to resolve microscopic neuronal characteristics of brain areas at a resolution beyond the fMRI spatial resolution. This approach involves the use of a paired stimulus paradigm in which two stimuli are successively presented within a short time interval, leading to equivalent improvement in fMRI spatial resolution^2^.

Several studies have suggested that this suppression phenomenon mostly occurs via passive processing of stimuli through a bottom-up (or feedforward) mechanism of signal processing. For example, repetition suppression of firing rates in the inferior temporal cortex (IT) has been observed in awake and anesthetized macaques^3^,^4^. Other studies have demonstrated that a period of 100–200 ms of repetition suppression in the visual ventral areas is too short to be mediated via top-down modulation because the mean population latency is approximately 150 ms in IT and below 75 ms in TE^4^,^5^. Furthermore, no repetition suppression effects of perceptual expectation have been observed in the lateral occipital complex (LOC) of human and monkey IT^6^,^7^.

However, several previous studies have shown that repetition suppression is modulated by perceptual expectation in some category-specific areas such as the fusiform body area (FBA), fusiform face area (FFA), and parahippocampal place area (PPA)^8^,^9^,^10^. Repetition suppression occurring in these studies is caused by top-down (or feedback) signal processing because it is modulated by different expectation probabilities, showing increased repetition suppression during high expectation probability and decreased repetition suppression during low expectation probability^11^,^12^. However, these studies did not examine some systematic details of the suppression mechanism related to perceptual expectation, including the temporal range over which expectation probability can effectively suppress the neuronal response to the stimulus or the high-level areas involved in repetition suppression and how they interact with the low-level areas within the network (i.e., through functional or effective connectivity).

In this study, we examined the systematic details involved in the top-down modulation of perceptual expectation using the repetition suppression phenomenon. Some previous studies have shown that repetition suppression is attributable to bottom-up mechanisms^3^,^4^ and the suppression disappears with intervals shorter than 1,000 ms^13^. Therefore, we used short and long inter-stimulus intervals (ISIs) in a perceptual expectation paradigm of paired stimulation, as described previously^11^, with the exception that we used ISIs of 250 and 1,000 ms and a short stimulus duration of 50 ms in the present study, in contrast to ISI of 500 ms and stimulus duration of 250 ms used previously (Fig. 1; Methods). In addition, we used effective functional connectivity analysis to investigate the causality between brain areas involved in repetition suppression.

## Results

Right fusiform face area (FFA_R) was individually identified from all participants by contrasting the response to a face to that to a scene in the localization run (x = 37.06 ± 4.96, y = −49.76 ± 9.86, z = −15.22 ± 5.26; p < 0.05, FDR-corrected) (Fig. 2).

For the main experimental runs, deconvolution analysis was performed because these runs involved the use of rapid event-related stimulation^14^. Estimated beta values were used for response comparison. The responses of the identified FFA_R to four trial conditions [60% repetition trials (Rr), 20% alternation trials (Ra), 20% repetition trials (Ar), and 60% alternation trials (Aa); Methods] were acquired after deconvolution of the data for the trials with 250 ms or 1,000 ms ISI. For trials with 250 ms ISI, the responses to Rr, Ra, Ar, and Aa were evaluated using two-way repeated measured (RM) ANOVA with block and trial factors. There was a significant interaction between these two factors (F_(1, 10)_ = 27.76, p < 0.001). Simple effect analysis revealed that Ra was significantly larger than Rr (pairwise comparisons, p < 0.001). However, there was no significant difference between Aa and Ar (pairwise comparisons, p = 0.50) (Fig. 3). To examine whether this difference was from response suppression at Rr or response enhancement at Ra, we compared Ra and Aa (i.e., the condition with high probability and the pair consisting of two different stimuli, therefore, it can be used as a reference value for evaluating signal changes caused by the effects of probability or repetition). Ra was larger than Aa (pairwise comparisons, p < 0.01). In addition, we compared Aa and Rr by paired t-test to examine whether Rr was suppressed or not. The comparison between Rr (i.e., the condition with high probability and the pair consisting of two identical stimuli) and Aa revealed a trend toward repetition suppression at Rr (Rr < Aa; paired t-test, p = 0.10). This indicates that strong response enhancement occurred and that it was dependent on stimulus probability. For trials with 1,000 ms ISI, no significant interaction (F_(1, 10)_ = 0.08, p = 0.78) and no significant main effects of the block and of the trial were found (F_(1,10)_ = 1.05, p = 0.33 and F_(1, 10)_ = 0.25, p = 0.63, respectively) (Fig. 3). This indicates that enhancement or repetition suppression did not occur for the trials at 1,000 ms ISI; those had been recovered during the interval. Although left fusiform face area was identified from only 5 participants and was not further analyzed for the main experiment, but the response pattern was similar to that of FFA_R (see Supplementary Figs S2 and S3).

To find other brain areas showing significant responses to stimulus trial conditions, we performed whole brain analysis only for 250 ms ISI trials because there was no difference among trial conditions for 1,000 ms ISI. The analysis revealed several areas in the occipital, temporal, parietal, and frontal lobes (p < 0.001, FDR-corrected) (Fig. 4). For each of those areas, two-way RM ANOVA was performed with block and trial factors, and a significant interaction was found at three areas: the left inferior frontal gyrus (IFG_L) (F_(1,10)_ = 21.44, p < 0.001) (Fig. 5A), left parietal gyrus (IPL_L) (F_(1,10)_ = 6.07, p < 0.05) (Fig. 5B), and left fusiform gyrus (FG_L) (F_(1,10)_ = 5.50, p < 0.05) (Fig. 5C). Therefore, simple effect analyses were performed after the ANOVA. Rr was significantly smaller than Ra at IFG_L, IPL_L, and FG_L (pairwise comparisons; p < 0.01, p < 0.001, and p < 0.05, respectively), indicating the same block effect as that at FFA_R. In addition, at IFG_L, Ra was larger than Aa (pairwise comparisons, p < 0.001), with no significant difference between Rr and Aa (paired t-test, p = 0.69). This indicated that probability-dependent response enhancement occurred at this brain area. At IPL_L, Rr was smaller than Aa (paired t-test, p < 0.05), with no significant difference between Ra and Aa (pairwise comparisons, p = 0.07). This indicated that probability-dependent repetition suppression occurred at in this brain area. At FG_L, Ra was larger than Aa (pairwise comparisons, p < 0.01), with no significant difference between Rr and Aa (paired t-test, p = 0.96). This indicated that probability-dependent response enhancement occurred at this brain area.

Time courses for the four areas showing repetition suppression were also evaluated. Because the expectation of a stimulus precedes the physical stimulus, the expectation-related activation of neuronal circuits may occur at relevant brain areas prior to the presentation of the physical stimulus^15^,^16^. Therefore, responses occurring at the stimulus onset (averaged signal intensity at the first time point of the time course across the trial condition) were evaluated for four brain areas with 250 ms ISI and 1,000 ms ISI. For 250 ms ISI trials, the response at IPL_L was significantly larger than the baseline (beta value = 0) (one-sample t-test; p < 0.001), while those at other areas were not (one-sample t-test; p = 0.33, 0.63, and 0.20 at IFG_L, FFA_R, and FG_L, respectively). One-way RM ANOVA revealed that responses at stimulus onset were significantly different among the four areas (F_(3, 30)_ = 4.53, p < 0.01). The response at IPL_L at onset was significantly larger than the response at FFA_R (p < 0.01). Compared with IFG_L, and FG_L, IPL_L at onset also showed a trend toward a larger response (p = 0.07 and p = 0.09, respectively; multiple comparison by Tukey’s test) (Fig. 6). The greater response at IPL_L preceding the stimulus indicates that the neuronal response at IPL_L precedes those at other areas and is not stimulus-induced but rather related to perceptual expectations. In addition, the effects of perceptual expectation on response enhancement and repetition suppression were observed in the repetition block but not in the alternation block. Therefore, the earlier response at IPL_L may reflect the modulation of other areas, particularly FFA_R, by IPL_L, which may explain why response modulation occurs in the repetition block. For trials with 1,000 ms ISI, there was no significance difference (F_(3, 30)_ = 1.77, p = 0.17). These results indicate that only for 250 ms ISI, expectation-related neuronal processing for response enhancement and repetition suppression initiated at IPL_L before the start of neuronal stimulus processing at FFA_R, IFG_L, and FG_L. This reflects a temporal relationship among these four areas for the stimulus event and these areas are considered to constitute a dynamic network (Fig. 7).

It is considered that the brain has to maintain a steady state on the basis of previous information gathered in the experimental run; the brain has to continuously count previous events and memorize them in rest periods as well as stimulation periods. To show that these four areas constitute a network, we assessed whether there existed a network related to steady-state neuronal processing by examining the whole time series using GCM among the four brain areas (IFG_L, IPL_L, FFA_R, and FG_L) for the experimental runs with 250 ms ISI. There was a strong positive influence on IPL_L from IFG_L and FG_L, i.e., there was effective connectivity from IFG_L and FG_L to IPL_L (p < 0.05, corrected) (Fig. 7; Supplementary Fig. S1). In addition, there was a strong positive influence on FFA_R from IFG_L, i.e., there was effective connectivity from IFG_L to FFA_R (p < 0.05, corrected) (Fig. 7; Supplementary Fig. S1). These findings indicate that these four areas constitute a network with two types of network modes: a dynamic network mode and a steady-state network mode.

Comparison of the behavioral performance of target detection showed that there was no significant difference in detection times for target trials between the repetition block and the alternation block (t-test, p = 0.28 for 250 ms ISI; see Supplementary Fig. S4), which were consistent with the previous paper^11^.

## Discussion

This study examined the top-down modulatory effects of perceptual expectation. We found modulatory effects of response enhancement and repetition suppression and examined related systematic factors: the high-level areas involved in the modulation, the temporal window range over which the expectation probability is effective for the modulation, and the interplay between high-level and low-level areas. We observed response enhancement and repetition suppression in the experimental run with 250 ms ISI and identified four brain areas in the frontal, temporal, and parietal regions that are involved in the response modulation with dual network modes: steady-state and dynamic network modes. We observed that the temporal window for effective response modulation ends up, at best, at 1,000 ms.

Although the motivation for this study was to examine the top-down modulatory mechanism related to repetition suppression using a paradigm similar to that used in a previous study^11^, we observed unexpectedly strong response enhancement at FFA_R with weak repetition suppression, strong response enhancement at IFG_L and strong repetition suppression at IPL_L.

For 250 ms ISI trials, the response enhancement and repetition suppression were observed only in the repetition block but not in the alternation block. This result is consistent with that of a previous study that had used the same probability-controlled paradigm and supported the top-down modulation hypothesis (perceptual expectation) for repetition suppression^11^. However, our results of the present study revealed response enhancement as well as repetition suppression.

In our results, the response enhancement may be explained as a top-down modulation similar to an odd-ball effect. FFA_R, IFG_L, and FG_L showed that the Aa response was smaller than the Ra response. Ra was an unexpected trial compared with Aa; therefore, an odd-ball-like effect may be observed in the Ra condition. In addition, alternation trials were not novel trials in the alternation block, which means that there was no chance for an odd-ball-like effect to occur. In the alternation block, an odd-ball-like effect could occur in repetition trials, but there may also be repetition suppression, which may lead to responses of similar magnitude during Ar and Aa.

On the other hand, another previous study reported repetition suppression with 200 ms ISI using a passive experimental paradigm in which the stimuli were repeatedly presented for just short flashes (several tens of milliseconds); thus, the suppression was considered to be attributed to bottom-up modulation^17^. Together with results of previous studies, the response enhancement and repetition suppression observed in the present study demonstrate that response modulation at FFA_R can be partly attributed to both top-down and bottom-up modulation. This modulation should have occurred not only during the repetition block but also during the alternation block if the response enhancement and repetition suppression had exclusively resulted from bottom-up modulation^11^,^12^. In this response modulation, IFG_L and IPL_L appear to work differently, i.e., IFG_L is more related to response enhancement and IPL_L is more related to repetition suppression, as shown in the response patterns of those areas (Fig. 5). It has been reported that IPL_L is involved in a goal-directing function, which causes repetition suppression in human studies^18^,^19^,^20^ or the prediction repetition priming phenomenon^21^.

Strong response enhancement and weak repetition suppression at FFA_R may be attributable to the stimulus parameter used. The current and previous study differ in terms of the stimulus duration of face pictures^11^. Although that difference could affect our results, it also demonstrates interplay of top-down and bottom-up processes in the response modulation.

For 1,000 ms ISI, unlike for 250 ms ISI, response enhancement and repetition suppression were not observed during repetition or alternation blocks. The finding that response modulation was observed with 250 ms ISI trials and not with 1,000 ms ISI trials suggests that, for our experimental conditions, the perceptual expectation effect on modulation faded when ISI lasted up to 1,000 ms, providing information regarding the temporal range over which this top-down modulation is effective. Previous studies^11^,^12^ have reported the attenuation of repetition suppression at 500 ms ISI; the present study shows response modulation at 250 ms ISI and disappearance of the modulation at 1,000 ms ISI, indicating that the disappearance would have occurred between 500 ms and 1,000 ms. The use of three or more ISIs in a future study would give more precise temporal information.

IFG_L, IPL_L and FFA_R showed response enhancement or repetition suppression at 250 ms ISI during just the repetition block. This indicates that response modulation results from the interplay between FFA_R and these high-level areas. This type of interplay is supported by previous studies that demonstrated the inferior frontal and parietal cortices to be involved in probability-related processing^22^,^23^,^24^. Therefore, through the identification of the involvement of the high-level areas IFG_L and IPL_L, the present study indicates that response enhancement and repetition suppression are attributable to top-down modulation more directly than previous studies did and suggests a brain network related to perceptual expectation^11^,^25^. Additional studies are needed to clarify whether the roles of the areas in this network are invariant regardless of the specific stimuli, tasks, or probabilities, which will give more information for understanding the interaction between low-level and high-level areas in this phenomenon.

Our results pertaining to the proposed brain network provide some information regarding the neuronal mechanism involved in top-down modulation signal processing. The earlier response at IPL_L in comparison with those at FFA_R, IFG_L, and FG_L indicates that top-down modulation is initiated first in the parietal area. Because response enhancement and repetition suppression are thought to be modulated by perceptual expectation, the early response at IPL_L may reflect a processing strategy in which stimulus probability information is updated first in the parietal area and is subsequently transferred to FFA_R, IFG_L, and/or FG_L for every stimulus input, constituting a dynamic network^26^,^27^. For this processing strategy to work, the parietal area has to maintain information regarding the frequency of previous trial occurrences or acquire this information from other areas.

Supporting that processing strategy is the steady-state network determined using GCM, which shows the directions of causality from IFG_L to IPL_L. This interaction between IFG_L and IPL_L in the steady-state network may be related to several cognitive functional processes such as attention, working memory, and predictive coding, and IFG_L seems to play some significant role in these processes. Many studies have shown that the frontal and parietal areas play a critical role in attention tasks, regardless of task types^28^,^29^,^30^, and that they create a fronto–parietal network^31^,^32^,^33^. The fronto–parietal network is also reportedly involved in working memory processing^34^,^35^. Furthermore, there are some studies showing that the fronto–parietal network is involved in familiarity manipulation via a predictive coding model^26^ as well as predictive modulation^36^,^37^. In addition to those previous reports, our result demonstrates that there was no significant difference in the response times for target trials between the repetition block and the alternation block in the ISI experiment (see Supplementary Fig. S4), which were consistent with the previous study^11^. These indicate that the responses in the higher areas cannot be explained by only a simple attentional effect and, therefore, suggest that IFG_L and IPL_L constitute a sub-network in the steady-state network and perform probability processing related to perceptual expectation.

Therefore, the results of previous studies and the present study suggest a mechanism for top-down modulation. In particular, the network composed of FFA_R, IFG_L, and IPL_L, operates in two modes: 1) The fronto–parietal interplay of IFG_L and IPL_L, working as a sub-network in the steady-state network mode, is involved in attending visual stimuli and sustaining information such as the probability variation in the task for the information for probability or expectation. 2) The information in the fronto-parietal sub-network is transferred to FFA_R from IPL_L in the dynamic network mode (Fig. 7).

Unlike other studies using explicit cues, the temporal relationship here is thought to result from an implicit processing mechanism of probability or expectation because we did not provide any explicit information to participants regarding expectation processing^38^,^39^,^40^,^41^. The similarity in behavioral data from trials with 250 ms ISI and 1,000 ms ISI also supports the implicit processing. This suggests that the paradigm used in the present study and in previous studies is useful to prove dynamic properties of brain networks^40^,^41^,^42^.

For the response in the parietal area, which is locked to the stimulus input prior to other areas, a signal may be required to the parietal area from an area earlier than FFA_R. This could arise through a ventral to dorsal pathway that carries event information such as the stimulus-onset information that triggers expectation information. One of the candidate pathways is a major white-matter fascicle (vertical occipital fasciculus) connecting dorsal and ventral visual cortices^42^. However, further study is needed for elucidating the existence of such a pathway.

With regard to the specificity of top-down modulation, the response enhancement and suppression observed at FFA but not at the other areas in the temporal lobe indicates that this type of top-down modulation is spatially specific to stimulus contents. This is supported by previous studies focusing on repetition suppression modulated by perceptual expectation. In a study involving monkeys, there was no repetition suppression effect in LOC by repetition probability^7^; however, there was a significant effect in FBA^9^, FFA, and PPA^8^.

As for the lateralization for top-down modulation to the left hemisphere, previous several studies provide a possibility of the involvement of the left hemisphere (IFG_L and IPL_L) in temporal orienting (focusing to a specific moment in time)^43^, disengaging temporally presented sequences of stimulus input^44^ and categorization of stimulus inputs^45^ although further study is required to clarify it.

From the physiological point of view, the activation of the parietal area at stimulus onset may not be consistent with the BOLD theory in which activation signals begin to rise at 2–4 s^46^,^47^. However, this type of response pattern is not unusual. In instances where prior information regarding a stimulus (such as a cue) had been given, a BOLD response was observed from stimulus-related areas before the responses to the physical stimuli appeared^38^,^39^,^40^,^41^. Although we did not provide any explicit cue in the present experiments, the expectation for probability variation may work as an implicit cue and activate the area prior to the activation by the physical stimulus.

Taken together, our results more directly verify that top-down modulation is involved in response enhancement and repetition suppression related to perceptual expectation and provide important systematic details for top-down modulation.

## Methods

### Participants

Eleven subjects (22.82 ± 0.55 years, four males and seven females) participated in the study. None of the subjects took medications that may affect brain function, and none of them had clinical or pathological neuropsychiatric disease. All subjects gave written informed consent. All experiments were performed in accordance with the relevant guidelines and regulations of the Gachon Gil Hospital’s Institutional Review Board, which approved all the experimental protocols.

### Experimental Procedure

**Figure 1:**
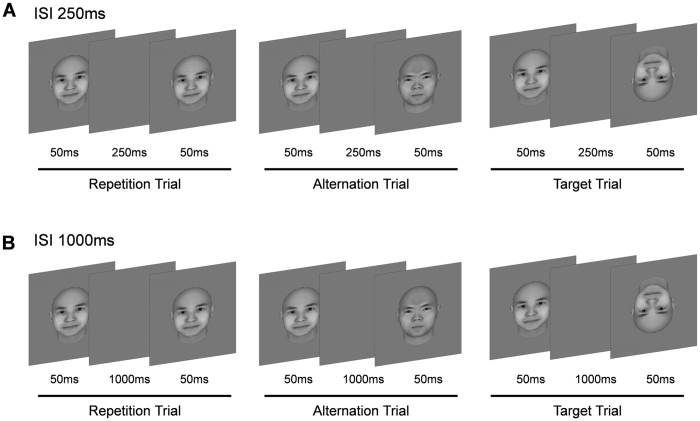
Experimental design. There were three types of trials: repetition, alternation, and target trials. Two different ISIs ((**A**) 250 ms and (**B**) 1,000 ms) were used for paired visual stimuli. The on time of the image was 50 ms.

**Figure 2:**
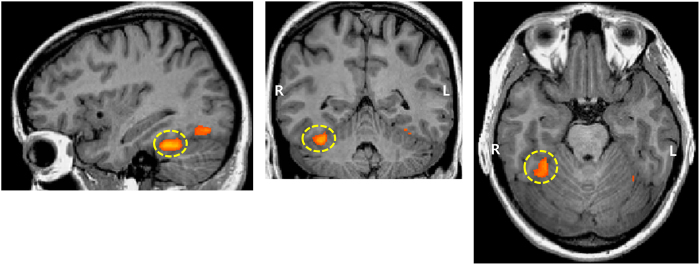
An example of an activation map for FFA_R. FFA (x = 37.06 ± 4.96, y = −49.76 ± 9.86, z = −15.22 ± 5.26, Talairach coordinates) was identified in the right hemisphere by comparing responses to face versus scene stimuli (p < 0.05, FDR-corrected).

**Figure 3:**
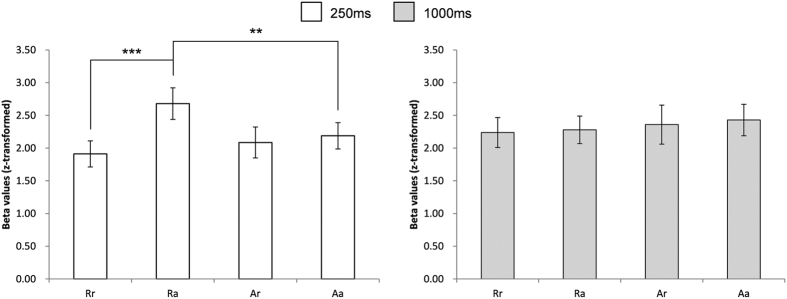
Responses during the four trial types (Rr, Ra, Ar, and Aa) in FFA_R for trials with 250 ms ISI and 1,000 ms ISI. ***p < 0.001, **p < 0.01. Error bars refer to standard error of the mean (SEM).

**Figure 4:**
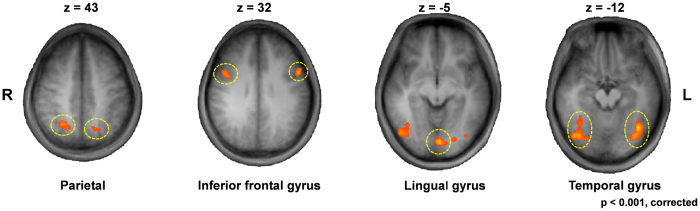
Activation areas of whole brain analysis based on estimated response using deconvolution analysis (p < 0.001, corrected). The location of these areas was represented by the following Talairach coordinates at peak statistical values: the right inferior frontal gyrus (39, 5, 31), left inferior frontal gyrus (IFG_L; −51, 8, 31), right parietal gyrus (27, −58, 43), left parietal gyrus (IPL_L; −12, −61, 46), lingual gyrus (−3, −79, −5), right temporal gyrus (42, −70, −11), and left temporal gyrus (FG_L; −39, −58, −11).

**Figure 5:**
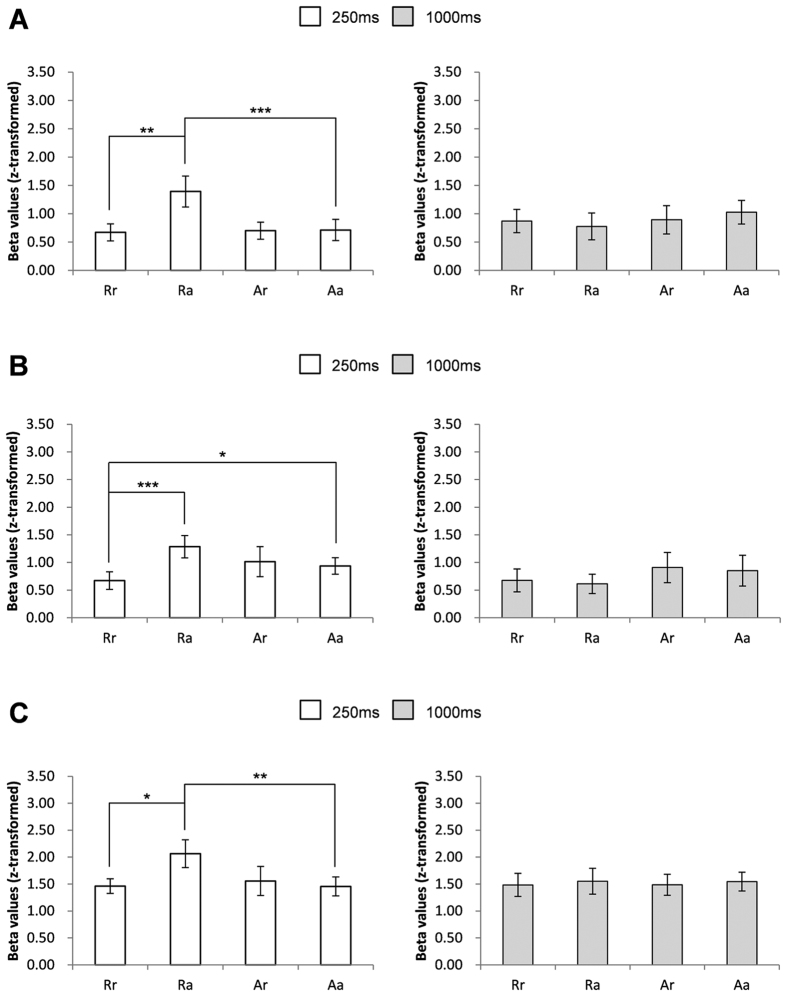
Responses during the four trial types (Rr, Ra, Ar, and Aa) in (**A**) IFG_L, (**B**) IPL_L and (**C**) FG_L for trials with 250 ms ISI and 1,000 ms ISI. ***p < 0.001, **p < 0.01, *p < 0.05. Error bars refer to standard error of the mean (SEM).

**Figure 6:**
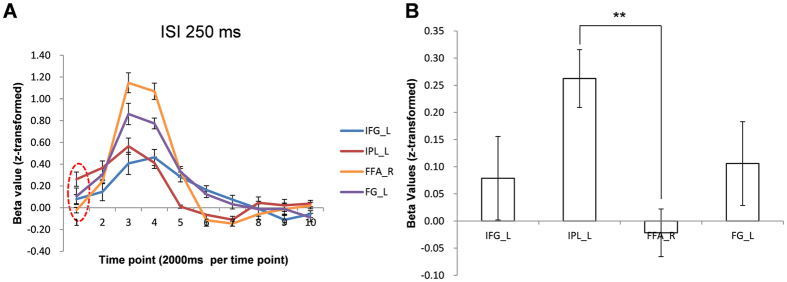
(**A**) Estimated time courses at FFA_R, IPL_L, IFG_L, and FG_R for trials with 250 ms ISI. (**B**) Responses at the stimulus onset in each area (IPL_L > FFA_R, p < 0.03; IPL > IFG_L, p = 0.07; IPL_L > FG_L, p = 0.09). **p < 0.01, Error bars refer to standard error of the mean (SEM).

**Figure 7:**
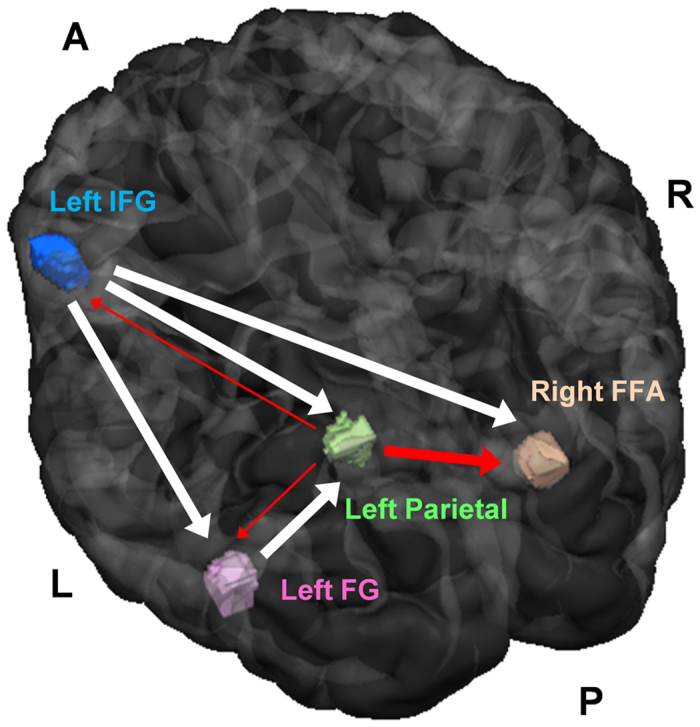
Three-dimensional view of dual network modes. White connections show the steady-state network mode, and red connections show the dynamic network mode.

A mixed block design, containing an event-related design in the stimulus block, was used (Fig. 1). There were three trial conditions: repetition, alternation, and target. Each trial consisted of facial images presented as paired stimuli separated by ISI. These images were generated using FaceGen (Singular Inversions, Toronto, Canada). The repetition trials used a pair of same images. The alternation trials used a pair of different facial images. The target trials used a pair of images in which the second facial image was an inverted version of the first image. The on-time for each image was 50 ms.

The study consisted of two task runs with different ISIs (250 ms and 1,000 ms) for each participant. Each task run was mixed block/event-related design. There were two kinds of blocks (repetition blocks and alternation blocks) and three kinds of trials (repetition trial, alternation trial and target trial). Repetition trial consisted of two same face images whereas alternation trial consisted of two different face images. In addition, target trial consisted of upright and inverted face images. Each block consisted of 20 trials including repetition, alternation and target trials. There were 12 repetition trials, 4 alternation trials and 4 target trials in each repetition block while 4 repetition trials, 12 alternation trials and 4 target trials in each alternation block. Repetition and alternation blocks were repeated thrice in each task at random order run and trials were separated by varying inter-trial intervals (2,000 ms–4,000 ms, mean = 3,000 ms). Three kinds of trial were presented randomly in both the repetition block and the alternation block. Each trial consisted of two stimuli with 50 ms duration and inter-stimulus interval of 250 ms, which made 350 ms for the 250 ms ISI experiment, and 1100 ms for the 1000ms ISI experiment. The stimulation was apparently event-related but implicitly grouped as the same way as the previous paper^11^. Each repetition and alternation block was 87 s.

To identify FFA, an additional experimental run was performed using a simple box-car design that included face and scene images. The run consisted of two types of stimulus blocks with either faces or scenes, which were interspersed with rest blocks. Each stimulus block was 12 s long and consisted of eight images. The duration of each image was 1500 ms. The rest block was 18 s long, during which a black crosshair was presented at the center of the screen on a gray background. Each stimulus block was repeated four times during the experimental run. The total scan time was 594s.

### Data Acquisition

The experiment was conducted using a 3-Tesla MRI scanner (MAGNETOM VERIO, Siemens, Erlangen, Germany). Functional images were obtained using a Gradient Echo single-shot echo planar image sequence (repetition time = 2,000 ms; echo time = 30 ms; in-plane resolution = 3.4 × 3.4 mm^2^; slice thickness = 3.4 mm; number of slices = 34 with no gap; slice orientation along AC-PC). Using a gradient echo, T1-weighted anatomical images were acquired with inversion recovery and magnetization-prepared rapid acquisition (repetition time = 1,900 ms; echo time = 2.93 ms; in-plane resolution = 1 × 1 mm^2^; slice thickness = 1 mm). Functional images and T1-weighted images had the same orientation for better co-registration in 3D-space.

### General Linear Model (GLM) Analysis

Functional image data were analyzed using Brainvoyager QX (Brain Innovation B.V., Maastricht, the Netherlands). Functional images were preprocessed by slice-timing correction, head motion correction, and temporal high-pass filtering (above 0.01 Hz) to remove physiological noise. These images were co-registered with each anatomical image. For analyzing our rapid event-related design, deconvolution analysis, which was implemented using Brainvoyager software, was performed. In deconvolution analysis, 10 shifted predictors per stimulus trial condition were used to define the respective BOLD responses. Each time point was 2,000 ms (one single volume), and a whole BOLD response (from the initial to final point) was considered to last for 20 s. GLM analysis was performed after deconvolution analysis. To acquire maximum estimated beta values, we used the third and fourth time points of the averaged estimated response (6 and 8 s after the stimulus onset) for random effect analysis in four trials (Rr, Ra, Ar, Aa)^48^. All beta values were corrected for serial correlation by autocorrelation and normalized by z-transformation.

### Granger Causal Model (GCM) Analysis

We defined IFG_L, IPL_L and FG_L as reference ROIs. All time-series from those ROIs were used in GCM analysis. GCM analysis was conducted by a plugin in Brainvoyager QX. We firstly ran GCM analysis individually with those reference ROIs. A causality map of each reference ROI was computed. The causality map was difference GCM (dGCM) which showed effective connectivity from the reference ROI to other brain areas. The dGCM represented two types of causalities. One was the connectivity from a reference ROI to other areas and the other was the connectivity from the other areas to the reference ROI. All dGCM of four reference ROIs were analyzed individually and group dGCMs were estimated by using one-sample t-test. Group dGCMs were corrected for multiple comparisons at a cluster-level threshold of p < 0.05 49.

## Additional Information

**How to cite this article**: Choi, U.-S. *et al*. Steady-state and dynamic network modes for perceptual expectation. *Sci. Rep.* **7**, 40626; doi: 10.1038/srep40626 (2017).

**Publisher's note:** Springer Nature remains neutral with regard to jurisdictional claims in published maps and institutional affiliations.

## Supplementary Material

Supplementary Information
