# Supplementary material for: Steady-state and dynamic network modes for perceptual expectation
Source: Sci Rep. 2017 Jan 12;7:40626. doi: 10.1038/srep40626 (PMC5228187; doi:10.1038/srep40626)
Supplement: Supplementary Information [file srep40626-s1.pdf]

Steady-state and dynamic network modes for perceptual expectation

Uk-Su Choi[1], Yul-Wan Sung[2*], Seiji Ogawa[1, 2]

[1] Neuroscience Research Institute, Gachon University of Medicine and Science, Incheon, Republic of Korea

[2] Kansei Fukushi Research Institute, Tohoku Fukushi University, Sendai, Japan

Correspondence:

Yul-Wan Sung, PhD

Kansei Fukushi Research Institute

Tohoku Fukushi University

Sendai, Japan

sung@tfu-mail.tfu.ac.jp; sungstone@gmail.com

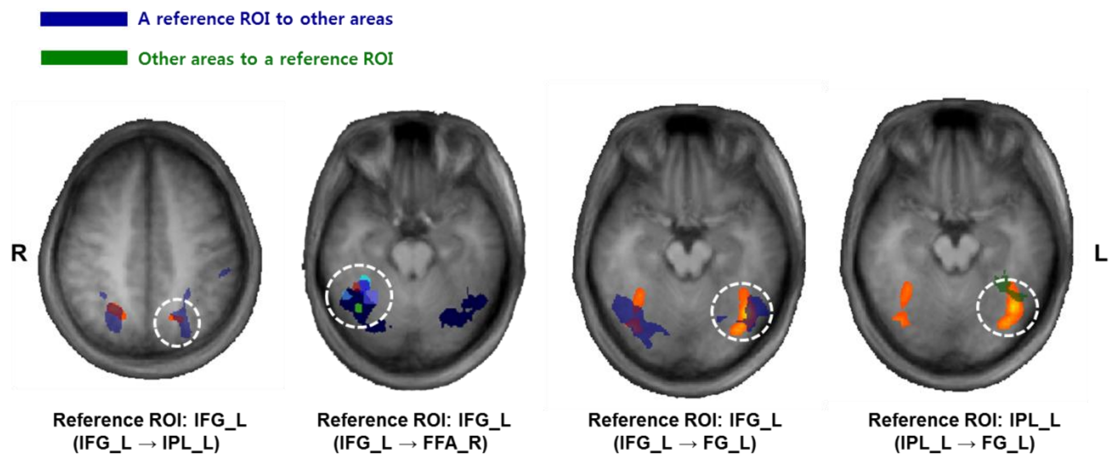

Supplementary Fig. S1 Overlay of the effective connectivity maps and four brain areas related to the task. Blue color stands for the effective connectivity from a reference ROI to the other brain areas and green color stands for the effective connectivity from the other brain areas to a reference ROI. Orange color stands for the task-related maps. White dashed circles indicate IFG_L, FFA_Rs (individual ROIs) and FG_L.

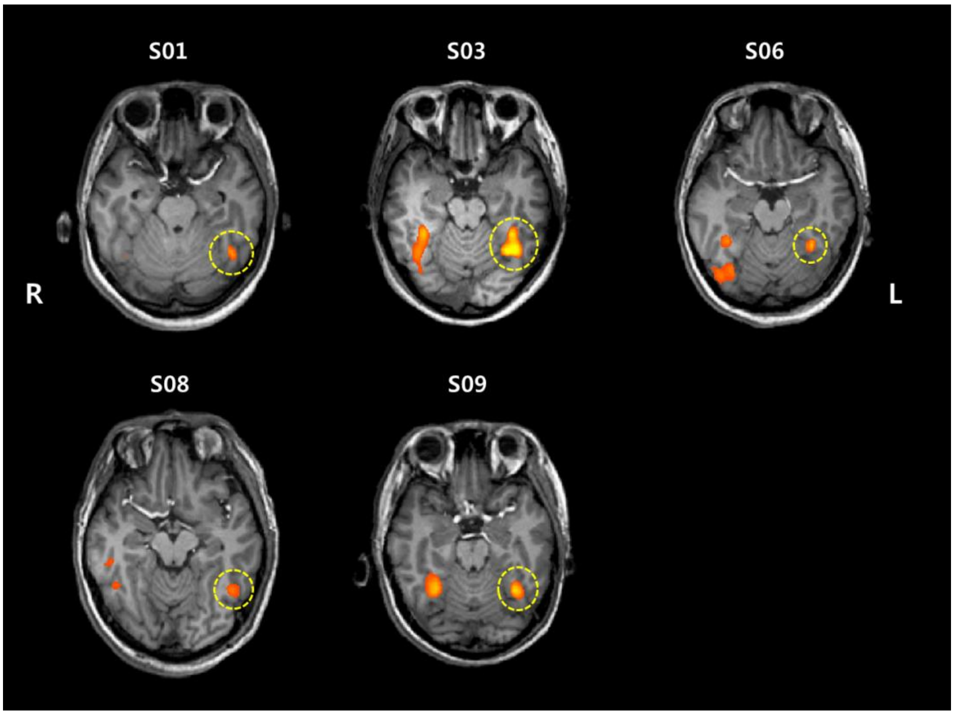

Supplementary Fig. S2 Individual left fusiform face area (FFA_L) localization. FFA_L was identified from only 5 participants ($p < 0.05$, FDR-corrected).

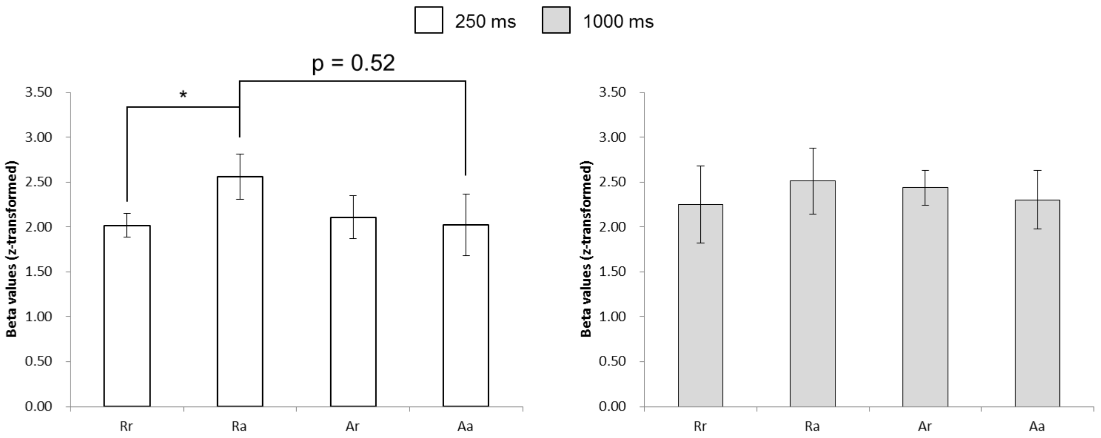

Supplementary Fig. S3 Response patterns of the four trial types (Rr, Ra, Ar, and Aa) in FFA_L. The left is for 250 ms ISI and the right is for 1,000 ms ISI. Error bars refer to standard error of the mean (SEM). *: $p < 0.05$,

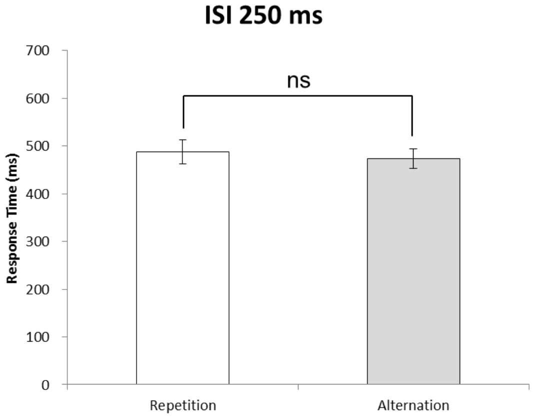

Supplementary Fig. S4 Response times for target trials of repetition block and alternation block in ISI 250 ms. Error bars refer to standard error of the mean (SEM). ns = not significant.
